# The MISOPRED score: Development and validation of a clinical scoring system to predict the effectiveness of Misoprostol treatment for early pregnancy loss

**DOI:** 10.1371/journal.pone.0303607

**Published:** 2024-05-31

**Authors:** Tomer Bar-Noy, Ofer Limonad, Erika Gandelsman, Alon Shrim, Hila Sharabi, Raphy Zarecki, Mordechai Hallak, Ilan Bruchim

**Affiliations:** 1 Department of Obstetrics and Gynecology, Hillel Yaffe Medical Center, Hadera, Israel; 2 School of Computer Sciences, Tel Aviv University, Tel Aviv, Israel; University of Brescia: Universita degli Studi di Brescia, ITALY

## Abstract

**Background:**

Misoprostol treatment for early pregnancy loss has varied success demonstrated in previous studies. Incorporating predictors in a single clinical scoring system would be highly beneficial in clinical practice.

**Objective:**

To develop and evaluate the accuracy of a scoring system to predict misoprostol treatment outcomes for managing early pregnancy loss.

**Study design:**

Retrospective cohort and validation study.

**Methods:**

Patients discharged from the gynecologic emergency department from 2013 to 2016, diagnosed with early pregnancy loss, who were treated with 800 mcg misoprostol, administrated vaginally were included. All were sonographically reevaluated within 48–72 hours. Patients in whom the gestational sac was not expelled or with endometrial lining >30 mm were offered a repeat dose and returned for reevaluation after seven days. A successful response was defined as complete expulsion. Clinical data were reviewed to identify predictors for successful responses. The scoring system was then retrospectively evaluated on a second cohort to evaluate its accuracy. Multivariate logistic regression was performed to identify factors most predictive of treatment response.

**Results:**

The development cohort included 126 patients. Six factors were found to be most predictive of misoprostol treatment effectiveness: nulliparity, prior complete spontaneous abortion, gestational age, vaginal bleeding, abdominal pain, and mean sac diameter, yielding a score of 0–8 (the MISOPRED score), where 8 represents the highest-likelihood of success. The score was validated retrospectively with 119 participants. Successful response in the group with the lowest likelihood score (score 0–3) was 9%, compared with 82% in the highest likelihood score group (score 7–8). Using the MISOPRED score, approximately 15% of patients previously planned to receive misoprostol treatment can be referred for surgical management.

**Conclusions:**

MISOPRED score can be utilized as an adjunct tool for clinical decision-making in cases of Early pregnancy loss. To our knowledge, this is the first scoring system suggested to predict the success rate in these cases.

## Introduction

Early pregnancy loss (EPL), including missed, incomplete, and complete abortion is the most common complication of pregnancy detected by transvaginal ultrasonography (TVUS) during the first trimester. EPL is estimated to occur in more than one-third of pregnancies and affects nearly 1 in 4 women [1, 2]. According to the American College of Obstetricians and Gynecologists (ACOG) the general definition of EPLis a nonviable, intrauterine pregnancy that is represented by an embryo or fetus that does not exhibit cardiac activity within the first 12-6/7 weeks of gestation or an empty gestational sac [3, 4]. When spontaneous EPL is diagnosed, management may include either medical treatment or surgical evacuation. Expectant management may be advised as well, especially in the presence of bleeding. To provide adequate counseling for patients, one must be aware of the traumatic experience for the patient, be empathetic and discuss all treatment options [4]. For women interested in expediting the process to complete expulsion and avoiding surgical evacuation, medical treatment with misoprostol, a prostaglandin E1 analog, may be advised [5]. The safety and effectivity of various methods for administering misoprostol have been studied. According to Cochrane reviews, oral administration was less effective than vaginal administration (RR 3.00, 95% CI 1.44 to 6.24), and may be associated with more frequent side-effects such as nausea and diarrhea. Similar effectivity was found for sublingual and buccal routes compared to vaginal administration, but they demonstrated higher rates of side-effects, mostly abdominal pain [6, 7]. Vaginal administration of 800 μg was found to be more effective than lower doses [8].

Drug administration may be a more convenient treatment approach than surgical evacuation for the patient and is associated with reduced hospital expenses and shorter hospital stays [9, 10]. Despite the high prevalence of misoprostol administration, it is still difficult to predict the risk of treatment failure using clinical or ultrasound criteria [11]. Previous studies have reported disparate success rates for medical treatment, ranging from 13% to 94% [12]. The uncertainty and variance of these rates may be due to a lack of parameters to predict the success of medical treatment [13]. Hence, the development of a predictive methodology and tool, for determining treatment effectiveness according to patient’s certain characteristics, indicative of favorable medical responses, would be highly valuable in clinical practice. This approach would integrate patient variables, ultrasound findings, and other pertinent parameters to gauge the likelihood of adverse outcomes, thereby facilitating informed clinical decision-making.

The current study proposes a clinical scoring system that was developed to predict the effectiveness of misoprostol treatment for EPL.

## Materials and methods

### Study setting and patient selection

A clinical scoring system for predicting the efficacy of misoprostol treatment for EPL was developed and validated based on retrospective data from an urban, tertiary care, academic medical center, that serves approximately 450,000 citizens.

The data were obtained from the electronic medical records of the Gynecologic Emergency Department. All patients ≥18 years of age with a singleton pregnancy <13 weeks’ gestation (as determined by last menstrual period (LMP)) and had TVUS performed either in the Gynecologic Emergency Department or the departments’ ultrasound unit, admitted with a diagnosis of EPL according to the ACOG guidelines and criteria for transvaginal ultrasonographic diagnosis of pregnancy failure and treated with 800 mcg misoprostol, vaginally, from January 2013 through December 2016 were included.

Patients with a known chronic disease, coagulation disorder, allergy to misoprostol, suspected ectopic pregnancy, trophoblastic disease, moderate or severe vaginal bleeding, incomplete abortion, pelvic infection, unstable hemodynamic state, or sepsis were excluded. Data recorded for each patient included demographics, gynecological and obstetric history (maternal age, gestational age based on LMP, number of previous pregnancies, abortions, and deliveries, including cesarean sections).

Patients were managed according to the following protocol. At the first visit to the ED (study day 1), a general and gynecological examination was performed, followed by TVUS to confirm the diagnosis of EPL and to estimate gestational age (GA). Patients were treated with 800 mcg misoprostol inserted vaginally into the posterior fornix. On study day 3, patients returned for reevaluation including TVUS, as accepted according to department protocol. A second dose of misoprostol was given if an intact sac or intrauterine content >30 mm was seen [14, 15]. On study day 9, patients returned for follow-up and an additional TVUS was performed.

Misoprostol treatment failure was defined by the presence of a gestational sac in the uterine cavity or residual endometrial thickness of more than 30 mm by TVUS, as accepted according to department protocol and according to the American College of Obstetricians and Gynecologists clinical management guidelines at the time the research was conducted [16]; Surgical evacuation was offered in case of medical treatment failure.

The study was approved by the Institutional Ethics Committee (Protocol #0102-18-HYMC from 30-Sep 2018). Informed consent was not required due to the retrospective nature of the data collection.

### Data

From an original set of 1,800 records, 245 patients met the inclusion criteria. Among these, 126 comprised the development cohort that was used to determine the variables to be incorporated in the prognostic scoring system. The validation cohort, intended to confirm the prognostic power of the model in a separate sample included 119 patients. Patients were randomly assigned to each group, all patient data was randomized and coded in separate database files prior to analysis.

### Deriving the scoring system

All variables of interest were analyzed using logistic regression, with estimated prevalence and odds ratios (OR) with 95% confidence intervals. Multivariate logistic regression was performed, in which we used forward selection and fivefold cross-validation to determine an appropriate model. The model incorporated analysis of two measures of prediction accuracy: misclassification rate and area under the receiver operating curve (AUC). By combining the observations, we generated the most consistent approximations of the coefficients for the model and subsequently developed a corresponding scoring system [17]. The variables that were integrated into the final multivariate model were separated into essential categories, each of which had a discrete value. A referent risk factor was subsequently selected for each factor and the associated risk was allocated a baseline score of 0; i.e., the higher the score, the higher the probability of treatment success. Following that, we determined the differences in regression units between each category and the related baseline category. We defined the constant, *b*, as the number of regression units that corresponds to 1 point. Next, we computed the score of the risk categories associated with each risk factor as the difference between the regression units in each category and the associated base category divided by the constant *b*. Subsequently, we used the outcomes of the multiple regression equations to allocate the risk ascribed to each point.

### Data stratification

Before the data validation stage, we developed the scoring system from the development data as described above, defining a 0 to 8-point scale. We also stratified this scale based on predicted clinical utility into low (9%), moderate (56%), and high (82%) probability levels of efficiency. We entered all factors from the development cohort and assigned point values of 0–8 and a risk category to every patient in the validation cohort.

### Data analysis

Categorical variables were analyzed using chi-square test and continuous variables were analyzed using independent *t* test or ANOVA, as appropriate. For all tests, a statistically significant *p*-value of less than 0.05 was defined. All analyses were performed using Microsoft Excel 2016 with Analysis ToolPak.

Six determinants were used to create the scoring system. These were identified as predictors because they showed significant differences between subjects. When using logistic regression, 10 events are required for each independent aspect of a clinical prediction [18]. As such, we were able to incorporate a maximum of 10 elements in a rule to calculate the need for intervention, while being sufficiently powered to develop a rule that delineated the most likely outcome. Including more than 10 elements would have reduced the performance, feasibility and accessibility of the scoring system that was developed [19].

### Sample size and power calculation

Medical management for EPL with misoprostol has a reported success rate of approximately 84% after a second dose [20].

## Results

A total of 245 patient records were included and analyzed in the study. Of these, 28% were primigravidas and 18% had had a previous cesarean section. The mean GA ± SD by LMP of all cases was 8.9 ± 1.7 weeks. Before receiving misoprostol, 22% of the patients had lower abdominal discomfort and 31% had vaginal bleeding. 61% experienced an abortion after the initial dose of misoprostol and 16% after the second dose, for an overall success rate of 77%. Those who experienced medical treatment failure underwent surgical evacuation. No statistically significant difference was found between failed and successful cases regarding the presence or absence of a fetal pole. Successful cases had significantly younger mean GA compared with failed cases (8.7 ± 1.7 vs. 9.9 ± 1.4 weeks, respectively; *p* = 0.003), parity was significantly lower (1.27 ± 1.2 vs. 2.05 ± 1.3; *p* = 0.008) as well, and lower abdominal pain correlated to a successful response to misoprostol (*p* = 0.04). Mean sac diameter measured by TVUS was significantly smaller in successful cases (11.3 ± 11.1 mm) as compared to failed cases (20.9 ± 11.7 mm; *p* = 0.003; Table 1).

**Table 1:** Characteristics of the development and validation cohorts.

Characteristic	Development cohort (n = 126)	Validation cohort (n = 119)
Mean age, years (±SD)	32.9 (±6.0)	33.1 (±6.4)
Primigravida	40 (31.7%)	30 (25.2%)
Multigravida	86 (68.2%)	89 (74.8%)
Mean gestational age by LMP, weeks (±SD)	9 (±1.5)	8.57 (±1.9)
Mean sac diameter, mm (±SD)	13.8 (±12.3)	11.5 (±10.9)
Vaginal bleeding upon diagnosis of EPL	40 (31.7%)	35 (29.4%)

### The MISOPRED score

Multivariate logistic regression analysis revealed 6 factors that were most significantly correlated with the efficacy of medical treatment: nulliparity, prior spontaneous complete abortion, younger GA, vaginal bleeding, abdominal pain, and smaller mean sac diameter (Table 2). These were incorporated into the MISOPRED score with associated point values, generating a total score ranging from 0 to 8. The higher the score, the greater the probability of success of misoprostol treatment.

**Table 2:** Predictors and scores for predicting likelihood of response to misoprostol.

Predictor	Category	Odds Ratio	Points
Parity	>2	1	0
	2	1.9	1
	<2	6.5	2
Previous abortion(s)	No	1	0
	Yes	2.1	1
Gestational age <7 weeks	No	1	0
	Yes	1.8	1
Vaginal bleeding	No	1	0
	Yes	1.7	1
Abdominal pain	No	1	0
	Yes	1.7	1
Mean sac diameter (mm)	>35	1	0
	25–35	2	1
	<25	5.1	2

### Retrospective validation

After developing the scoring system, we assessed its performance using two standard measures: calibration and discrimination. Calibration was evaluated by developing a plot of the proportions of events that were detected versus the projected likelihoods, as demarcated by the range of individual predicted risks. Distinguishing events versus non-events was defined by discrimination measured by the AUC [21–23].

The multivariate logistic regression model had a misclassification rate of 0.25 (95% confidence interval [CI] 0.22–0.28) and an AUC of 0.84 (95% CI 0.80–0.88), whereas the MISOPRED score had a misclassification rate of 0.26 (0.25–0.27) and an AUC of 0.82 (0.74–0.90). Agreement between the risk estimates based on the MISOPRED score and those based on the multivariate logistic regression model had a *κ* constant of 0.85 (95% CI 0.84–0.86), showing insignificant loss of accuracy by assigning whole number scores to the predictors.

The statistical analysis indicated that the quantitative effects of the 6 factors included in the MISOPRED score can accurately predict treatment outcomes and allow stratification of subjects into groups of probability of success: low (0–3 points), moderate (4–6 points) or high (7–8 points) (Table 3).

**Table 3:** Probability and estimated response according to MISOPRED score.

Probability	Total points	Estimated response (%)
Low	0–3	9–55
Moderate	4–6	56–81
High	7–8	82–100

## Discussion

There are three acceptable approaches to the management of EPL: expectant management, medical treatment, and surgical evacuation. Individualization and treatment “tailoring” should be performed, taking into consideration various aspects, including patient preferences, GA, comorbidities, gynecological and obstetrical issues. Although the medical treatment approach is considered highly safe and effective, the current study approached an important clinical question troubling many patients and physicians—can we predict in advance which patient will have a “high chance” of success with misoprostol treatment, and which patients are more likely to experience treatment failure. By addressing these questions, we can select and suggest the most suitable treatment approach for each patient.

To accomplish this, we developed a clinical scoring system, termed MISOPRED, for the prediction of misoprostol treatment success for EPL. We analyzed the clinical and ultrasonographic variables correlated with the effectiveness of misoprostol treatment and derived the MISOPRED score. Then, we retrospectively evaluated the extent to which this scoring system would have improved medical treatment outcomes in patients with EPL.

The study was conducted in two steps. First, the most predictive variables were derived from the development cohort, which was followed by validation of the model on a different cohort. Six factors that were most predictive of successful treatment: nulliparity, prior complete spontaneous abortion, younger GA, vaginal bleeding, abdominal pain, and smaller mean sac diameter were found.

The effectiveness of misoprostol depends mainly on GA: the earlier the GA, the higher the success rate. This result is in accordance with a study that found a trend toward decreased effectiveness in women at 10 to 12 weeks of gestation, as compared with those at 9 weeks or less, using the same dose of misoprostol [24]. Similarly, higher parity was negatively associated with the success rate.

In addition, sonographic parameters exhibited strong clinical and statistical effects in predicting the effectiveness of the medical treatment. Smaller mean sac diameter and smaller fetal poles were associated with increased success rates after the first or second dose.

A significant problem with many studies is the definition of success. In a prospective randomized study that included 809 women undergoing first-trimester uterine evacuation, 18 had retained products of conception [25]. Evaluation of the 8 mm cut-off for predicting retained products of conception demonstrated a sensitivity of 100% and a specificity of 99.1%.

Another study included 97 women who complained of residual vaginal bleeding 15 days or more after taking oral misoprostol for EPL. The results of the sonographic scan correlated with the histopathological examination of uterine contents. Endometrial thickness ^3^12 mm predicted incomplete abortion with a sensitivity of 88.5% and specificity of 73.7% [26, 27]. Conversely, other studies reported that gestational sac volume did not predict successful treatment, and no obvious relationship between increasing endometrial thickness and the need for surgical intervention could be established [28].

Recent data indicated that combined treatment with misoprostol and mifepristone is more effective than treatment with misoprostol alone for first and second trimester abortions [29], however data regarding safety and side effects seems to be equivocal in Kelesidou et al. recent review of the literature [30]. Nevertheless, the misoprostol-alone regimen is considered an acceptable treatment according to all guidelines, and is used in several treatment protocols, including in our department, and is considered the treatment of choice in settings in which mifepristone is not available, mostly due to the differences in socioeconomics in different parts of the world [31, 32]. It should be noted that at more advanced GA, our treatment protocol adds mifepristone to misoprostol, as well. In recent years, the effectiveness of misoprostol for EPL has been documented in several studies. Creinin et al. reported that misoprostol treatment is gaining acceptance from physicians and gives patients the opportunity to decrease the time until expulsion and avoid surgical interventions. They reported that the success rate of misoprostol is 71% (95% CI 67–75%) after one dose and 84% (95% CI 81–87%), overall. Therefore, 16% of patients will experience treatment failure and be referred for surgical management [33]. Thus, it has become increasingly important to be able to identify patients at high-risk of misoprostol treatment failure. To date, there is no reliable tool that can incorporate all known predictive factors. Scoring systems are usually generated to assist with clinical decision-making. The concept is practical because it enables the clinician to turn qualitative judgment into quantitative assessment [34].

The three-level stratification of probability of successful treatment in the development and validation cohorts showed that approximately 20% and 21% were categorized as having a low chance, 70% and 67% as moderate, and 10% and 12% as high, respectively. The overall success rate among the development and validation cohorts was approximately 10% and 8% in the low probability group, 55% and 58% in the moderate group, and 80% and 84% in the high probability group, respectively. Hence, if a patient has a low probability, she is very likely to have a failed response. In such a case, medical treatment is not advisable, which means that a surgical approach would be more appropriate. If the clinical decision is to treat only patients in the high probability group (MISOPRED score >7) with misoprostol, then the expected success rate would be at least 82%, as compared with an overall 77% without using the scoring system. With such an approach among the low probability group, approximately 90% of patients could have avoided misoprostol treatment and an unfavorable outcome.

This study was limited by the relatively small sample size, which can lead to sampling bias. A possible solution for small sample size is to conduct collaborative studies instead of analyzing a single-center, retrospective cohort as was done here. Different centers may have other protocols for diagnostic workup, treatment choices, definition of predictors, etc. Therefore, larger collaborative, multicenter studies can increase the sample size and improve the accuracy of the model.

In addition, the current study is retrospective in nature, with the possible biases of this methodology. These included missing data that might have affected the final predictors of the score or that patients who were excluded because of incomplete records, might have different predictors. Therefore, we are currently conducting a follow-up, prospective validation study, to examine the use of the MISOPRED scoring system in terms of validity, reproducibility and statistical accuracy.

The strengths of this study are that it demonstrates practical applicability and high accuracy in predicting the likelihood of a good response to misoprostol. Most notably, to our knowledge this is the first study to incorporate the best predictors identified, using machine learning methods, into a single clinical scoring system. We believe that this scoring system will revitalize the application of predictive research in gynecology, leading to an essential utilization in clinical practice. While we acknowledge the limitations of this model, we believe that we have established a novel scoring system that has the potential to be improved with additional data and to be very useful in practice. Our results provide compelling evidence for the long-term application of this model and suggest that the MISOPRED score appears to be effective.

The MISOPRED score presents an objective approach for both the clinician and the patient that could improve shared decision-making. We believe that such a scoring system can be incorporated into routine practice to improve treatment effectiveness and reduce adverse outcomes.

## Supporting information

S1 Data (XLSX)
